# The role of S-nitrosylation of PFKM in regulation of glycolysis in ovarian cancer cells

**DOI:** 10.1038/s41419-021-03681-0

**Published:** 2021-04-15

**Authors:** Wenwen Gao, Mengqiu Huang, Xi Chen, Jianping Chen, Zhiwei Zou, Linlin Li, Kaiyuan Ji, Zhirui Nie, Bingsheng Yang, Zibo Wei, Pengfei Xu, Junshuang Jia, Qianbing Zhang, Hongfen Shen, Qianli Wang, Keyi Li, Lingqun Zhu, Meng Wang, Shuangyan Ye, Sisi Zeng, Ying Lin, Zhili Rong, Yang Xu, Peng Zhu, Hui Zhang, Bingtao Hao, Qiuzhen Liu

**Affiliations:** 1 Cancer Research Institute, Guangdong Provincial Key Laboratory of Cancer Immunotherapy, Guangzhou Key Laboratory of Tumor Immunology Research, School of Basic Medical Sciences, Southern Medical University, Guangzhou, 510515 China; 2 Southern Hospital Zengcheng Branch, Southern Medical University, Guangzhou, 528308 China; 3 First Affiliated Hospital of Zhengzhou University, Zhengzhou University, Zhengzhou, Henan Province 450001 China; 4 Guangzhou Panyu Central Hospital, Guangzhou, 511400 China; 5 Pearl River Hospital, Southern Medical University, Guangzhou, 528308 China; 6 Henan Cancer Hospital, Zhengzhou University, Zhengzhou, Henan Province 450003 China; 7 Guangzhou Concord Cancer Center, Guangzhou, 528308 China; 8 The Eighth Affiliated Hospital, Sun Yat-sen University, Shenzhen, Guangdong 518033 China; 9 Central Lab of Shenzhen Pingshan People’s Hospital, Shenzhen, 518118 P. R. China; 10 School of Biomedical and Pharmaceutical Sciences, Guangdong University of Technology, Guangzhou, 510006 China; 11 Metabolic Innovation Center, Zhongshan School of Medicine, Sun Yat-sen University, Guangzhou, Guangdong 510080 P. R. China; 12 Platform of Metabolomics, Center for Precision Medicine, Sun Yat-sen University, Guangzhou, Guangdong 510080 P. R. China; 13 Medical Genetic Institute of Henan Province, Henan Provincial Key Laboratory of Genetic Diseases and Functional Genoics, Henan Provincial People’s Hospital Zhengzhou University People’s Hospital, Henan University People’s Hospital, Zhengzhou, Henan 450003 China; 14 Pingshan General Hospital of Southern Medical University, Southern Medical University, Shenzhen, 518118 China

**Keywords:** Cancer metabolism, Nitrosylation

## Abstract

One of the malignant transformation hallmarks is metabolism reprogramming, which plays a critical role in the biosynthetic needs of unchecked proliferation, abrogating cell death programs, and immunologic escape. However, the mechanism of the metabolic switch is not fully understood. Here, we found that the S-nitrosoproteomic profile of endogenous nitrogen oxide in ovarian cancer cells targeted multiple components in metabolism processes. Phosphofructokinase (PFKM), one of the most important regulatory enzymes of glycolysis, was S-nitrosylated by nitric oxide synthase NOS1 at Cys351. S-nitrosylation at Cys351 stabilized the tetramer of PFKM, leading to resist negative feedback of downstream metabolic intermediates. The PFKM-C351S mutation decreased the proliferation rate of cultured cancer cells, and reduced tumor growth and metastasis in the mouse xenograft model. These findings indicated that S-nitrosylation at Cys351 of PFKM by NOS1 contributes to the metabolic reprogramming of ovarian cancer cells, highlighting a critical role of endogenous nitrogen oxide on metabolism regulations in tumor progression.

## Introduction

The metabolic reprogramming intently engaged in the transformation and progression of tumors^1^. Cancer cells are characterized by an increased conversion of glucose to lactate in the presence of sufficient oxygen, known as the Warburg effect^2^. Glycolytic flux is tightly controlled in eukaryotic organisms by allosteric regulation of enzymes. Phosphofructokinase1 (PFK1), an enzyme catalyzing fructose-6-phosphate (F-6-P) to fructose-1,6-diphosphate (F-1, 6-BP), is a pacemaker for glycolysis^3^. The formation of tetramers is required for the functional activity of PFK1 (ref. ^4^). Many metabolites allosterically regulate equilibrium between dimer and tetramer^5^. The inhibitory allosteric metabolites such as ATP, lactate, and citrate inhibit tetramer formation and provide negative feedback for glycolysis rate^6^. The increase of nutrients uptake and accumulation of metabolic intermediates are indispensable for macromolecular synthetics in the aggressive proliferation of tumors^7^. Activation of PI3K-dependent AKT or AMPK induced phosphorylation of phosphofructokinase 2 (PFK2), which produces allosteric activator of PFK1 (F-2, 6-BP) to overcome negative feedback of glycolysis^8,9^. However, the mechanism of resistance to high metabolism intermediates in tumors remains to be further studied.

Nitric oxide (NO) is an essential metabolic regulator synthesized by three different NO synthases (NOS1, NOS2, and NOS3) from l-arginine and molecular oxygen^10^. The increased expression of NOSs predicts poor prognosis in various types of cancer^11^. NO-mediated biological events are mainly based on protein modification of S-nitrosylation (SNO-protein), the covalent attachment of the nitric oxide group to the thiol side chain of cysteine^12^. S-nitrosylation has been documented in multiple tumor biology processes, including unchecked proliferation, abrogate cell death programs, and immunologic escape^13–15^. Endogenous NO plays a critical role in metabolic rewiring towards glycolysis through inhibition of mitochondrial respiration^16^. S-nitrosylation of the AKT pathway components regulates the expression and activity of enzymes^17^. Ovarian cancer, one of the most aggressive gynecologic cancers, is characterized by high glycolysis^18^. Recently, NO has been reported to participate in the metabolic conversion of ovarian cancer^19^.

PFKM, an isoform of PFK1, is expressed mainly in normal muscle and neuron tissues. PFKM binds NOS1 through the PDZ domain^20,21^, enabling a rapid regulation of glycolysis in response to energy requirement in muscle and apoptosis resistance in neurons^22^. Dysregulation of the association of PFKM with NOS1 leads to muscle functional ischemia and damage^23^. NOS1 constitutively synthesizes a low level of NO and plays a critical role in tumor progressions^24^. The expressions of both NOS1 and PFKM increase in different kinds of tumors and positively correlated with the progressive stages of ovarian cancer. Here, we explored the role of PFKM S-nitrosylation modification induced by NOS1 on the metabolic switch in ovarian cancer.

## Materials and methods

### Cell line culture, plasmids, and reagents

The human ovarian cancer cell lines SKOV3, OVCAR3, A2780, and melanoma cells B16-F10 were purchased from the American Type Culture Collection (ATCC) and were routinely tested for absence of mycoplasma using a Mycoplasma Detection Kit (Bitool, China). All the cells were grown in DMEM medium (Gibco, Gaithersburg, MD, USA) supplemented with 10% fetal bovine serum (Biological Industries, Beit Haemek, Israel) and maintained at 37 °C in a humidified atmosphere of 5% CO_2_. Stable NOS1 over-expression (OE-NOS1) and nontargeted control (Cont) cell lines were generated according to a previously reported method^25^. We constructed an ovarian cancer cell line (SKOV3) that stably knocks out NOS1 or PFKM using CRISPR/Cas9 technology by the UCSC genomic database (http://genome.ucsc.edu/). We chose the sequences to design the gRNA of NOS1 or PFKM into the expression vector P2U6-pCAG-Cas9 and P2U6-Kana-Cas9 which were generously provided by Dr. Zhili Rong. After the recombinant plasmid was successfully constructed, P2U6-NOS1-KO-Cas9 or P2U6-PFKM-KO-Cas9 was transfected into ovarian cancer cell SKOV3 cells by liposome. Similarly, the plasmid P2U6-PFKM-KO-Cas9 corresponding to the mouse gene was transfected into B16-F10 cells. Multiple monoclonal cells were screened with puromycin, and then the protein expression was verified. Five successful clones were selected for further culture. After five generations of culture, the protein expression was verified, and two successfully knockout clones were selected for experiments. We constructed PFKM-WT and PFKM-C351S sequences were cloned into the expression vector pCDH-CMV-MCS-EF1-RFP which were designed and synthesized from Synbio Technologies (Suzhou, China). Then we successfully constructed stable cell lines PFKM-WT, PFKM-C351S, OENOS1+PFKM-WT, and OENOS1+PFKM-C351S on the SKOV3-PFKM-KO cells. At the same time, we used mouse gene sequences to construct stable cell lines PFKM-WT and PFKM-C351S using the expression vector pCDH-CMV-MCS-EF1-RFP on the B16-F10-PFKM-KO cells.

The chemicals GSNO and N-PLA were obtained from Cayman Chemical (Ann Arbor, MI, USA). Ascorbic acid (VC), citric acid, lactic acid, and ATP were purchased from Sigma Company (Sigma-Aldrich, St. Louis, MO, USA).

### Cell proliferation assay

Ovarian cancer cells were seeded in 96-well culture plates at a density of 2000 cells per well. Cell Counting Kit-8 (CCK8) (GK10001, GlpBio, America) was added to each well and incubated for 1–4 h. The absorbance was read at 450 nm using a spectrophotometer (BioTek, Winooski, VT, USA).

### Tumor and mice models

BALB/c-nu mice and C57BL/6 mice (Female, 6–8 weeks old) were all purchased from Guangdong Medical Laboratory Animal Center. They were then housed in SPF facilities on a 12-h light/dark cycle until the end of the experiment. All animal experiments in this study were approved by the Medical Ethics Committee of Southern Medical University. Injected into two different thigh roots of 10 mice (BALB/c-nu mice) and inoculated with ovarian cancer cells (5 × 10^6^ cells in 100 μl PBS) subcutaneously in the dorsal flank. We randomly divided 10 mice into two groups labeled A and B. We injected SKOV3-PFKM-WT into the left lower thigh of the rats in group A, and injected SKOV3-OENOS1 + PFKM-WT into the right lower thigh. We injected SKOV3-PFKM-C351S into the left lower thigh of the rats in group B, and injected SKOV3-OENOS1-PFKM-C351S into the right lower thigh. On day 15, tumor length (*L*) and width (*W*) were measured with a Vernier caliper every 5 days and tumor volumes were calculated using the formula *V* = (*L* × *W*^2^)/2. The animals were euthanized on day 35. The tumor tissues were excised and weighed.

C57BL/6 mice were randomly assigned to two groups of 15 mice each (labeled A and B, respectively). B16-PFKM-WT cells were injected into the tail vein of group A, and B16-PFKM-C351S cells were injected into the tail vein of group B. Mice were euthanized during days 15–30 post injection, and lung tissue was isolated, photographed, and then fixed with 4% formaldehyde for histological and morphometric measurements.

### Glucose consumption assay and lactate secretion assay

Cells were seeded in 96-well plates at a density of 5000 cells per well; the culture media were collected. Glucose Colorimetric Assay Kit II (#K686-100, BioVision) was used for glucose consumption assay according to the manufacturer’s instruction. Absorbance at a wavelength of 450 nm was determined using a spectrophotometer. Lactate Colorimetric Assay Kit II (#K627-100, BioVision) was used for lactate secretion assay according to the manufacturer’s instruction. Absorbance at a wavelength of 450 nm was determined using a spectrophotometer.

### Western blotting analysis

RIPA lysis buffer or PBS supplemented with the PMSF and phosphatase inhibitor cocktail (Beyotime, China) was used for total protein extraction. The intracellular protein was fully cleaved and sonicated by a ultrasonic sonicator (Q800R, Qsonica, America) for 15 min. Protein concentration was determined with BCA protein assay kit (#E162-01, KeyGen Biotech). Equal amounts of protein were separated by SDS-PAGE or non-reducing SDS-PAGE and transferred to a PVDF membrane (Millipore, Billerica, USA). The membrane was blocked with 5% bovine serum albumin and probed with the appropriate primary antibodies: rabbit anti-PFKM (55028-1-AP, 1:500 dilution) and rabbit anti-PFKP (13389-1-AP, 1:500 dilution) and rabbit anti-GST (10000-0-AP, 1:5000 dilution) and rabbit anti-FLAG Tag (20543-1-AP, 1:1000 dilution) were from Proteintech; rabbit anti-nNOS (ab76067, 1:1000 dilution) and rabbit anti-PFKL (ab241093, 1:1000 dilution) were from Abcam (Cambridge, UK). After incubating overnight at 4 °C, the membrane was then probed with horseradish peroxidase (HRP)-conjugated goat anti-rabbit IgG secondary antibody (Proteintech, USA). Signals were visualized using the eECL Western Blot Kit (#P90720, Millipore).

### EdU cell proliferation assay (imaging assay)

EdU Apollo 567 in Vitro Kit from Guangzhou Ruibo Company was used for cell proliferation detection according to the manufacturer’s instruction.

### Immunoprecipitation and co-immunoprecipitation analysis

Proteins were extracted from cultured cells using a modified buffer, followed by immunoprecipitation and co-immunoprecipitation (Co-IP) with the corresponding antibodies^26^. The assay was performed according to standard procedures.

### PFK1 activity

ATP consumed by the PFK enzyme is regenerated by PK, which converts phosphoenolpyruvate (PEP) into pyruvate. Pyruvate is then reduced to lactate by a NADH-dependent lactate dehydrogenase (LDH), and the consumption of NADH can be monitored spectrophotometrically at 340 nm. PFK protein was purified from ovarian cancer cells by immunoprecipitation (IP), or the whole protein of ovarian cancer cells were extracted for detection of PFK1 activity. The reaction was started by the addition of PFK and run at 37 °C in a 96-well plate with a reaction volume of 150 μl. The assay mix consisted of 50 mM Tris pH 9.0, 2.0 mM magnesium sulfate, 5.0 mM potassium chloride, 0.728 mM PEP, 5 mM F-6-P, 1.0 mM ATP, 0.5 mM β-NADH, 0.35 U PK, 0.5 U LDH, and appropriate amounts of PFK (see the “Result” section). We use the following formula to calculate the enzyme activity of PFK1: sample phosphofructokinase activity = *B*/(Δ*T* × *V*) = nmol/min/ml = mU/ml, where *B* is the NADH amount from standard curve (nmol), Δ*T* is the reaction time (min), and *V* is the sample volume added into the reaction well (ml).

### Pull-down assay

We recombinantly synthesized the peptide of NOS1-PDZ domain tagged with glutathione S-transferase (GST). GST pull-down assays were performed^27^. Briefly, glutathione agarose beads were incubated with GST-NOS1-PDZ and purified PFKM overnight. The beads were then washed with lysis buffer for five times.

### S-nitrosylation detection assay (IBP)

S-nitrosylated Protein Detection Assay Kit (Cayman, USA) based on the “Biotin-switch” method was used for S-nitrosothiol detection according to the manufacturer’s instruction. All steps were done with minimal light exposure. Briefly, 100–250 μg protein lysates were extracted from cells or the digested tumor tissues. Its free thiols were blocked by blocking agent. S-nitrosothiols in the protein were reduced to free thiol(s) and subsequently covalently labeled with maleimide-biotin. Then we purified all S-nitrosylated proteins using Streptavidin Agarose Resins. After concentration measurement and dilution with SDS loading buffer, the labeled proteins were denatured at 95 °C. The membrane was blocked with 5% bovine serum albumin and probed with the appropriate primary antibodies: rabbit anti-PFKM (55028-1-AP, 1: 500 dilutions). Samples were sent to immunoblotting with HRP detected reagent and visualized by the eECL Western Blot Kit (#P90720, Millipore). Western blot detection of the content of the SNO-PFKM which was enriched by streptavidin.

### liquid chromatography tandem mass spectrometry (LC-MS/MS) analyses the S-nitrosylation sites

The free cysteine thiols on the proteins were Salkylated by adding three volumes of blocking buffer (20 mM MMTS in HEN buffer plus 5% (w/v) SDS) to the protein mixture and incubating at 60 °C for 30 min in the dark^28^. The protein pellet was simultaneously reduced and labeled in HEN buffer containing 1 mM ascorbate solution, 4 mM Biotin-maleimide, and 1% SDS at room temperature for 2 h in the dark (Irreversible Biotinylation Procedures, IBP). Then we used trypsin to digest the protein, and the biotin peptides were enriched by Streptavidin Agarose Resins. The biotinylated peptides were eluted in 1 ml of avidin eluting buffer (30% ACN and 0.1% TFA or 8 M guanidine hydrochloride pH = 1.5) at room temperature for 10 min with vigorous agitation^29^.

The LC-MS was used for the detection of the Orbitrap Fusion three-in-one mass spectrometer. The ion source voltage was set to 2.4 kV, and the peptide parent ions and their secondary fragments were detected and analyzed using high-resolution Orbitrap. The primary mass spectrometer scan range is set to 350–1500*m*/*z*, the scan resolution is set to 120,000, and the Orbitrap scan resolution is set to 30,000. The data acquisition mode uses a data-dependent scanning (DDA) program, in which the peptide precursor ions enter the HCD collision cell sequentially and use 30% of the fragmentation energy for fragmentation after the first-stage scanning, and the secondary MS is sequentially performed. In order to improve the effective utilization of the mass spectrometer, the automatic gain control is set to 2E5, the signal threshold is set to 5E4 ions/s, the maximum injection time is set to 100 ms, and the dynamic exclusion time of the MS/MS scan is set to 30 s to reduce the parent ion.

Secondary mass spectral data were retrieved using Thermo Proteome Discoverer 2.1. The search parameters are set as follows: the database is set to human protein sequence or protein sequence of PFKM; the enzyme cut mode is set to Trypsin; the missed cut point number is set to 2; the primary parent ion mass error tolerance is set to 10 ppm; the secondary fragment ion mass error tolerance is set to 0.02 Da; variable modification is set to oxidize methionine, acetylation of N-terminus of the protein, biotinylation (Cys, +451.2 Da), alkylation, S-nitrosation of cysteine, MMTS (+45.9877 Da), carbamidomethyl (Cys), peptide ion requirements are not in Decoy Database Search, The identification result peptide confidence is set to high.

### Bioinformatics

Kyoto Encyclopedia of Genes and Genomes (KEGG) database was used to identify pathways using DAVID software.

### Generation of PFKM expression vectors for ovarian cancer cells expression

Flag-tagged WT human PFK1 (M isoform) cDNA (ATCC, NCBI accession# NP_000280.1) was cloned into the expression vector PCDNA3.1. The Flag-tagged C351S, Flag-tagged C553S, and Flag-tagged C631S PFKM mutants were generated using the QuikChange II Site Directed Mutagenesis Kit (Agilent Technologies).

The primers are as follows:

PFKM-WT-F 5′-tagggagacccaagctggctag-3′, PFKM-WT-R 5′-aaacgggccctctagactcga-3′;

PFKM-C351S-F 5′-tgcctctgatggaaagcgtcc-3′, PFKM-C351S-R 5′-aaacgggccctctagactcga-3′;

PFKM-C553S-F 5′-ccacttctgatcgcatcaaacag-3′, PFKM-C553S-R 5′-ctgtttgatgcgatcagaagtgg-3′;

PFKM-C631S-F 5′-cctgcgcaacgaaaagtctaacg-3′, PFKM-C631S-R 5′-cgttagacttttcgttgcgcagga-3′.

### Metabolite extraction

For analysis of metabolism, 50 × 10^4^ cells/well SKOV3-WT cells and SKOV3-C351S cells were seeded onto a six-well plate with basal medium in at least three repeats for 12 h, allowing cells attaching enough. Then steady-state labeling of organic was accomplished by changing the culture medium into glucose-free DMEM medium containing appropriate tracer, including 11 mmol/l U-^13^C_6_-Glucose, and the cell metabolites were extracted after 24 h of culture.

At the end of culture, cells were rinsed twice with normal temperature 0.9% NaCl solution, quenched with 500 μl/well −80 °C methanol. One minute later, 200 μl of ice-cold 5 μg/ml norvaline was added to each well and cells were collected in 1.5 ml Eppendorf tubes by scraping with a pipette. Then 500 μl of −20 °C chloroform was added to each tube followed by vortexing for 15–20 min, and centrifugation at 14,000 × *g* for 10 min at −4 °C. The upper aqueous phase and the lower organic layer were transferred to the fresh tube and exsiccated of airflow, respectively. These dried samples can be stored at −20 °C.

### Metabolite derivation and GC-MS analysis

Derivation should be done within 24 h before detection. Polar metabolites were derivatized to form methoxime–tBDMS derivatives by dissolved upper dried metabolites with 20 μl of 2% (m/v) methoxylamine hydrochloride in pyridine and incubating at 37 °C for 60 min. Samples were then silylated by addition of 100 μl of MTBSTFA with 1% tBDMS and incubated at 45 °C for 30 min. Transferred to glass GC vials for analysis.

The column was tested with TG-35 (30 m, 0.25 mm, 0.25 um) using an Agilent 7980A-GC/5975C-MS. A 1 μl sample was injected at 270° and a nitrogen flow rate of 1 ml/min was used, using a split mode to avoid sample overload. To separate the polar material produced by Mox-TBDMS, the chromatograph oven was set to 100° 2 min, increased to 255° at 3.5°/min, then increased to 320° at 15°/min, and maintained at 320° for 3 min. Electron impact ionization was operated with the MS scanning over the range 100–650*m*/*z*.

### Oxidative phosphorylation and glycolysis assays

The intact cellular oxygen consumption rate (OCR) and extracellular acidification rate (ECAR) were measured in real time using the Seahorse XF96 Extracellular Flux Analyzer (Seahorse Bioscience). Briefly, 1.0 × 10^4^ SKOV3-PFKM-WT or SKOV3-PFKM-C351S cells were seeded into 96-well Seahorse microplates in 80 μl of growth medium and incubated at 37 °C in 5% CO_2_ for 16 h and the calibrator plate was equilibrated overnight in a non-CO_2_ incubator. Before starting the test, cells were washed twice with assay running media (unbuffered DMEM, 25 mmol/l glucose, 1 mmol/l glutamine, 1 mmol/l sodium pyruvate for OCR; unbuffered DMEM, 1 mmol/l glutamine for ECAR) and equilibrated in a non-CO_2_ incubator. Once the probe calibration was completed, the probe plate was replaced by the cell plate. The protocol was optimized for the simultaneous measurement of OCR and ECAR. For OCR, the analyzer plotted the value as the cells were treated by sequential injection of the following compounds: oligomycin (1 μmol/l), carbonyl cyanide-4 (trifluoromethoxy) phenylhydrazone (FCCP, 0.5 μmol/l), and antimycin A (1 μmol/l) plus rotenone (1 μmol/l). For ECAR, the analyzer plotted the value as the cells were treated by sequential injection of the following compounds: glucose (10 mmol/l), oligomycin (1 μmol/l), and 2-deoxy-glucose (2-DG, 100 mmol/l).

### Flow cytometry

For analysis of tumor-infiltrating lymphocytes, resected tumor tissues were cut into small pieces and then digested in collagenase I (1 mg/ml) and 13.3 μl DNase I (50 U/ml) at 37 °C for 30 min. The mixture was filtered through a 70-um strainer to prepare a single-cell suspension. Cells were then washed twice with PBS and re-suspended in PBS, and 1 × 10^6^ cells were incubated with 3 μl antibody for 30 min at 4 °C in darkness. The cells were washed twice and performed the analysis on the FACSCalibur (BD Biosciences, USA). The anti-mouse CD3-PE-Cy7, CD8-FITC, CD45-APC-Cy7, F4/80-PE, CD25-PerCPCy5.5, CD4-Alexa Fluor 647, and CD11b-BV650 antibodies were all purchased from BD Biosciences. Data are represented as the percentage of lymphocytes from parents as indicated.

### Statistics

All values were presented as mean ± SD as experiments independently were carried out in triplicate. Data were analyzed using one-way or two-way ANOVA or Student’s *t*-test (GraphPad Software, Inc., San Diego, CA, USA), depending upon the number of groups and variances. Kaplan–Meier survival plots were compared using a log-rank test. The difference was considered statistically significant when the probability was less than 0.05 (*P* < 0.05).

## Results

### The key components in glycolysis pathway are highly S-nitrosylated in ovarian cancer cells

**Fig. 1:**
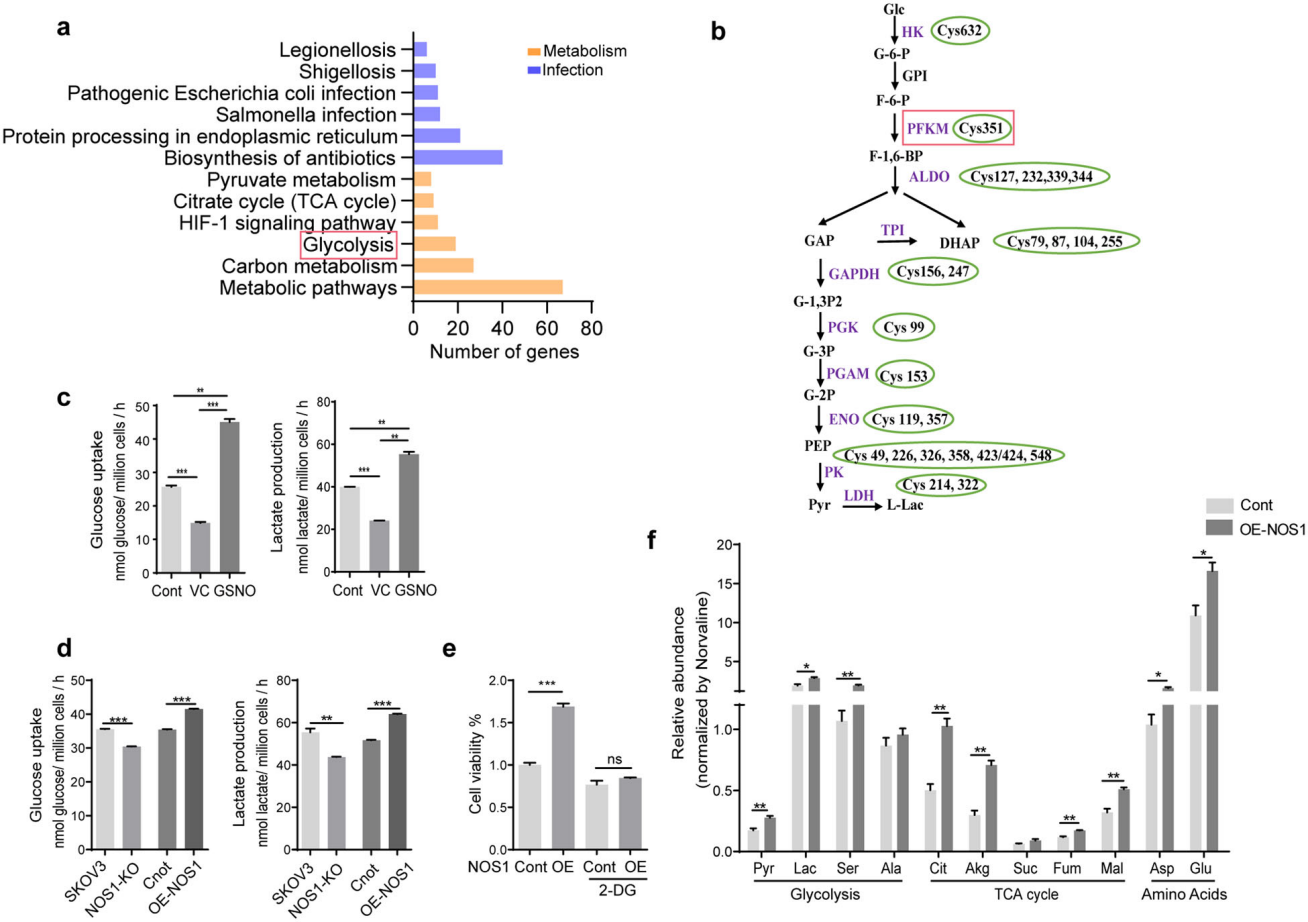
The key components in glycolysis pathway are highly S-nitrosylated in ovarian cancer cells. **a** The KEGG analysis for the significant enrichment pathways of 527 SNO proteins in SKOV3 cells (*n* = 4). **b** The S-nitrosylated sites of glycolytic enzymes in SKOV3 cells. **c** Glucose uptake and lactate production of SKOV3 cells treated with ascorbic acid (VC), GSNO, or PBS, respectively, at 37 °C for 30 min (*n* = 3). **d** Glucose uptake and lactate production of SKOV3-NOS1 knockout (NOS1-KO) vs. SKOV3 cells, and SKOV3-NOS1 over-expression (OE-NOS1) vs. SKOV3-control cells (*n* = 3). **e** Over-expression NOS1, control cells, and these SKOV3 cells treated with 2-DG (1 mM) for 48 h were subjected to CCK8 assay (*n* = 3). **f** Detect the total amount of glycolytic, TCA cycle intermediate synthesis, and amino acids in SKOV3-OE-NOS1 and control cells using GC-MS analysis technique. Each value represents the mean ± SD in three independent experiments. ns not significant; *P < 0.05, **P < 0.01, ***P < 0.001, Student’s *t-*test.

Nitric oxide affects the biological processes of cancer mainly through the S-nitrosation of targeted proteins^30^. We delineated the nitrosoproteome in ovarian cancer cell line SKOV3 cells by enriching the S-nitrosylated proteins with streptavidin agarose resins and identifying the protein through MS/MS. A total of 527 proteins containing 838 S-nitrosocysteine peptide fragments were obtained in SKOV3 cells. KEGG pathway analysis showed that these proteins are enriched in cellular metabolisms (carbon metabolism, glycolysis, citrate cycle, etc.) and infection-related pathways (*Salmonella* infection, pathogenic *Escherichia coli* infection, *Shigellosis*, etc.) (Fig. 1a). We found that most of the enzymes in glycolysis were modified by S-nitrosylation, suggesting that S-nitrosylation modification plays a critical role in regulating glycolysis (Fig. 1b and Table S1). The ALDOA-Cys339, GAPDH-Cys156, Cys247, PGK-Cys99, PGAM-Cys153, PKM-Cys326, Cys423, and Cys424 sites had been reported previously in human tissues or cells, while we did not find the other sites in the dbSNO 2.0 database. The PFKM-C351 site was reported to be S-nitrosylated in the myocardium of mouse, as shown in the database^31^. S-nitrosoglutathione (GSNO), an endogenous S-nitrosothiol (SNO), is generally used to mimic NOS-derived NO function. GSNO treatment increased the glucose consumption and lactate secretion (two indicators of glycolysis) in SKOV3 cells (Fig. 1c). The glucose consumption and lactate secretion were decreased in the cells treated with ascorbic acid (VC), a reducing agent inducing the denitrosation of proteins (Fig. 1c). These data indicated that NO selectively targeted the proteins involved in the metabolism processes, thereby regulating cancer cell glycolysis.

NOS1 synthesizes constitutively NO at a low level, and we have reported that NOS1 usually promotes the progression in kinds of cancers^32^. We constructed an ovarian cancer cell line (SKOV3) that stably deleted NOS1 using CRISPR/Cas9 technology. Two successfully knockout clones were selected for further experiments. To explore the role of NOS1 in glycolysis, we measured glycolysis in SKOV3-OE-NOS1 and SKOV3-NOS1-KO cells. The result showed that the glycolysis increased in SKOV3-OE-NOS1 cells and reduced in SKOV3-NOS1-KO cells, indicating that NOS1 is involved in regulating the glycolysis in cancer cells (Fig. 1d). Analysis of NOS1 expression in human ovarian cancer tissues from the GSE database (GSE14407) found that NOS1 expression in cancer tissues was higher than in normal tissues (Fig. S1a). The hierarchical clustering of a class of glycolysis-related genes distinctly separated the normal samples from the cancer samples, and NOS1 was clustered to the group of the glycolysis enzymes that were highly expressed in cancer. 2-Deoxy-d-glucose (2-DG) is a glycolysis inhibitor. Treatment with 2-DG attenuated the cell viability induced by NOS1 over-expression in SKOV3 cells (Fig. 1e). Similar results were obtained in OVCAR3 cells (not shown). Then we detected the relative abundance of glycolytic metabolites by using the gas chromatography-mass spectrometry (GC-MS) technique. As expected, the relative abundance of glycolytic metabolites (pyruvate, lactic acid, and serine), TCA cycle metabolites (citrate, α-ketoglutaric acid, fumaric acid, and malic acid), and amino acids (aspartic acid and glutamic acid) increased in SKOV3-OE-NOS1 cells (Fig. 1f).

PFK1, the first rate-limiting enzyme in the glycolytic pathway, is a key metabolic hub in glycolysis regulation. PFKM isoform was S-nitrosylated in our S-nitrosoproteome profile of ovarian cancer cells. So we investigated the connection of NOS1 with PFKM related to cancer cell proliferation. We found that PFKM knockout (PFKM-KO) dramatically slowed down the cell proliferation of SKOV3 cells (Fig. S1b). The effect of NOS1 on glycolysis and cell proliferation was weakened in PFKM-KO cells, indicating the NOS1’s function was dependent on PFKM (Fig. S1c, d). These results suggest that NOS1 can enhance the glycolysis and TCA cycle of cancer cells, promoting cancer cell proliferation.

### NOS1 enhances its activity through S-nitrosation of PFKM

**Fig. 2:**
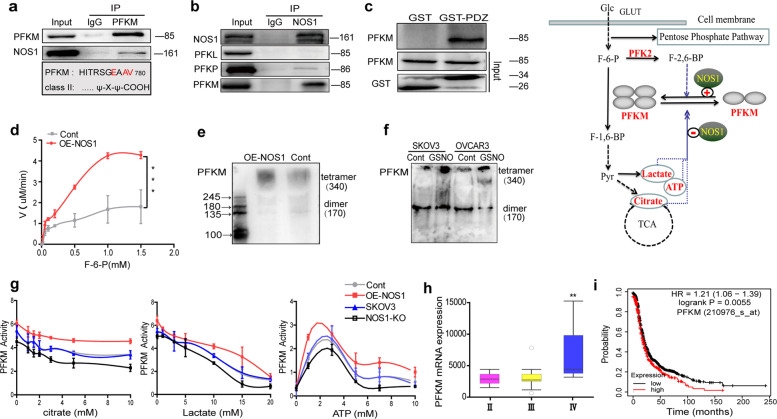
NOS1 enhances its activity through S-nitrosation of PFKM. **a** Co-immunoprecipitation (Co-IP) detected whether PFKM with NOS1 could bind in SKOV3-OE-NOS1 cells by using PFKM monoclonal antibody (upon); PFKM, one proposed NOS1 binding partner, display a class II PDZ domain-binding motif at its C termini (down). **b** Co-IP detected whether NOS1 with PFKL, PFKP, and PFKM could bind in SKOV3-OE-NOS1 cells by using NOS1 monoclonal antibody. **c** Western blot results showed that PDZ domain of GST-tagged could bind to PFKM by GST pull-down assay. **d** Enzyme kinetics assay displayed the *K*_m_ (the concentration of F-6-P required to reach half-maximal activation) decreased significantly and the *V*_max_ (maximum velocity) increased significantly in the SKOV3-OE-NOS1 cells (*K*_m_ = 0.591 and 0.692, *V*_max_ = 8.33 and 2.6 μM/min for OE-NOS1 and control cells respectively) (***P < 0.001, one-way ANOVA). **e** Western blotting by non-reducing SDS-polyacrylamide gel electrophoresis (SDS-PAGE) displayed the tetramer and dimer contents of PFKM in OE-NOS1 and control SKOV3 cells. **f** The tetramer and dimer contents of PFKM in SKOV3 and OVCAR3 cells treated with GSNO (1 mM) or PBS for 30 min respectively. **g** The activity of PFKM under different concentrations of citrate (left), Lactate (middle) and ATP (right) in SKOV3 vs. NOS1-KO cells and SKOV3-OE-NOS1 vs. control cells. **h** Boxplots correlating the stage of tumors and the mRNA expression levels of tumor glycolytic genes PFKM are shown in ovary cancer samples (GSE51373). (**p < 0.01, Student’s *t*-test). **i** Kaplan–Meier analyzed for the overall survival time between PFKM high or low expressed patients.

**Fig. 3:**
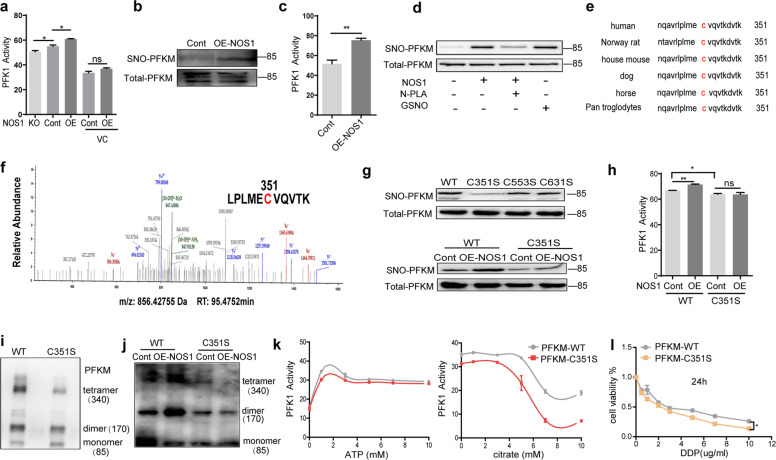
NOS1 induced S-nitrosylation of PFKM at Cys351. **a** The activities of PFK1 were measured in SKOV3-NOS1-KO, control, and SKOV3-OE-NOS1 cells vs. control and SKOV3-OE-NOS1 cells with ascorbic acid (VC) for 30 min (*n* = 3). **b** Western blot detected the content of SNO-PFKM protein in the control and OE-NOS1 tumor tissues which inoculated with SKOV3-OE-NOS1 and SKOV3-Cont cells. **c** The activities of PFK1 were measured in the SKOV3-control and SKOV3-OE-NOS1 tumor tissues (*n* = 3). **d** Biotin switch then purified all S-nitrosylated proteins in SKOV3 cells using streptavidin agarose resins. Western blot detected the content of S-nitrosation-modified PFKM (SNO-PFKM) protein in the SKOV3 cells, OE-NOS1 cells, the cells which were treated with GSNO (1 mM) for 30 min, and the OE-NOS1 cells treated with NOS1-specific inhibitor N-PLA (100 μM) for 48 h. **e** Amino acid sequence alignment of PFKM. The conserved cysteine residue (**c**, red) is S-nitrosated by NO. **f** Identification of the S-nitrosylation site on PFKM using mass spectrometry in SKOV3 cells. According to the data obtained by the mass spectrometer, the search database was searched by Thermo Proteome Discoverer 2.1. **g** Western blot detected the content of SNO-PFKM protein in SKOV3-PFKM-KO cells were transfected with Flag-PFKM WT or Flag-PFKM C351S, C553S, and C631S, respectively (upon). SKOV3-PFKM-KO cells and PFKM-KO+OENOS1 cells were transfected with Flag-PFKM-WT or Flag-PFKM-C351S, respectively. Western blotting detected the content of SNO-PFKM protein (down). **h** The enzymatic activity of PFK1 was detected in the SKOV3-PFKM-KO cells and PFKM-KO+OENOS1 cells which were respectively reconstituted with Flag-PFKM-WT or Flag-PFKM-C351S. **i** Western blot detected the contents of PFKM tetramer, dimer, and monomer in the SKOV3-PFKM-KO cells which were respectively reconstituted with Flag-PFKM-WT or Flag-PFKM-C351S. **j** Western blot detected the changes in PFKM tetramer, dimer, and monomer in the SKOV3-PFKM-KO cells and PFKM-KO+OE-NOS1 cells which were respectively reconstituted with Flag-PFKM-WT or Flag-PFKM-C351S. **k** Different concentrations of ATP (0–10 mM, left) or citrate (0–10 mM, right) were added to observe the activity of PFK1 from the total protein in SKOV3-PFKM-WT or SKOV3-PFKM-C351S cells (*n* = 3). **l** Cell viability% detected by CCK8 assay. Different concentrations of DDP were applied for 24 h in the SKOV3-PFKM-WT and SKOV3-PFKM-C351S cells, respectively (*P < 0.05 by one-way ANOVA).

We speculated the effect of NOS1 on the regulation of PFK1 activity. We found that GSNO treatment and over-expression of NOS1 significantly increased the total activity of PFK1 in SKOV3 and OVCAR3 cells (Fig. S2a and Fig. 3a). Similar results were obtained in A2780 cells (not shown). The previous reports showed that the PDZ ligand sequence of the NOS1 interacts with PFKM and protects neurons from apoptosis by the promotion of glycolysis. Using Co-IP assay, we confirmed the interaction between NOS1 and PFKM in SKOV3-OE-NOS1 cells (Fig. 2a, b). NOS1 has a weak interaction with PFKP and no interaction with PFKL (Fig. 2b). A GST pull-down assay using purified GST-NOS1-PDZ and purified Flag-PFKM showed that NOS1-PDZ bound to PFKM (Fig. 2c).

Therefore, to test whether NOS1 affected the kinetic correlation constant of PFKM in ovarian cancer cells, we measured the enzyme activity of PFKM at gradient substrate concentrations. Enzyme kinetics assay showed the conversion rate of fructose-6-phosphate to fructose-1,6-diphosphate catalyzed by the purified PFKM increased significantly in SKOV3-OE-NOS1 cells (Fig. 2d). The PFKM tetramer in SKOV3-OE-NOS1 cells is more than that in control cells (Fig. 2e). GSNO treatment also increased PFKM tetramer form in SKOV3 and OVCAR3 cells (Fig. 2f). To explore the alteration of PFKM tolerance to allosteric inhibition, we detected the activity of PFKM under gradient concentrations of metabolic inhibitors. Selective NOS1 inhibitor (N-PLA) decreased the relative PFKM activity under citrate, lactate, and ATP inhibition in SKOV3 cells (Fig. S2b). The enzymatic activity of purified PFKM from SKOV3-OE-NOS1 cells was higher than that from control cells in gradient concentrations of citrate, lactate, and ATP (Fig. 2g). Consistent with NOS1 over-expression, the PFKM from NOS1-KO cells had lower activity than that from SKOV3 cells (Fig. 2g). The tolerance of PFKM to allosteric inhibition was more significant under high concentrations of citrate (>5 mM) and lactate (>5 mM) (Fig. 2g). ATP has a dual effect on the activity of PFK1 in which the low level of ATP increased, and high-level ATP reduced the activity of PFK1 (ref. ^33^). Our experiment showed over-expression of NOS1 made PFKM more sensitive to low-level ATP activation and more tolerant to high-level ATP inhibition.

Three isoforms of PFK1 (PFKM, PFKL, and PFKP) can form hetero-tetramers^34^. We examined the total activity of PFK1 in cellular extracts and found that NOS1 over-expression also significantly enhanced the tolerance of PFK1 to citrate and ATP but not lactate in SKOV3 cells (Fig. S2c). We surveyed the clinical significance of the PFK1 isoform expression from public databases. PFKM expression was positively correlated with the progressive stages of ovarian cancer (GSE51373) (Fig. 2h). The ovarian cancer patients with high expression of PFKM experienced significantly shorter overall survival (Fig. 2i). These results suggested that NOS1 enhances the PFKM activity and its tolerance to allosteric inhibition of citrate and ATP.

### NOS1 induced S-nitrosylation of PFKM at Cys351

The content of S-nitrosylation of protein depends on the extent of both S-nitrosylation and denitrosylation^35^. In biological systems, SNO bond cleavage yields radical or ionic species on exposure to heat, light, reducing agents, or nucleophilic compounds^12^. Ascorbic acid (VC) treatment reduced the NOS1’s effect on the PFK1 enzyme activity in SKOV3 cells (Fig. 3a). Over-expression of NOS1 increased PFKM S-nitrosylation and the activity of PFKM in the xenograft tumors inoculated with SKOV3-OE-NOS1 and SKOV3-Cont cells (Fig. 3b, c). Moreover, treatment with GSNO or over-expression of NOS1 increased, but NOS1-specific inhibitor N-PLA reduced S-nitrosylation of PFKM in SKOV3 cells (Fig. 3d) and OVCAR3 cells (Fig. S3c).

We predicted three cysteine sites of PFKM (Cys351, Cys553, and Cys631) as potential S-nitrosylation modified sites using GPS-SNO 1.0 software. Among the three cysteine sites, C351 was located at the conserved region of PFKM and was reported as an S-nitrosylated site in mice (Fig. 3e). We confirmed the S-nitrosylation of Cys351 by using the LC-MS/MS technique in SKOV3 cells (Fig. 3f). We constructed plasmids of PFKM mutants with Cys351, Cys553, Cys631 mutated to serine, and transfected the PFKM mutant plasmids into PFKM-knockout SKOV3 cells. Only the cells transfected with Cys351 mutant (C351S) but not C553S and C631S displayed reduced S-nitrosylation of PFKM compared to PFKM-WT (Fig. 3g). Over-expression of NOS1 upregulated the S-nitrosylation level of PFKM-WT but not PFKM-C351S in SKOV3 cells (Fig. 3g). These data indicated that Cys351 of PFKM was the S-nitrosylation site by NOS1.

C351S mutation led to a slight reduction of PFKM activity and abolished NOS1’s regulation on PFKM activity (Fig. 3h and Fig. S3a). We explored the S-nitrosylation of PFKM on the equilibrium between dimers and tetramers using western blot using non-reducing SDS-polyacrylamide gel electrophoresis (SDS-PAGE). The tetramer and dimer of PFKM were more than that of monomer in PFKM-WT cells, while the C351S mutation reduced the tetramer and slightly increased monomer in SKOV3-PFKM-KO cells (Fig. 3i). NOS1 expression increased monomers’ transformation to tetramers and dimers of PFKM-WT protein, but not the PFKM-C351S mutant in SKOV3-PFKM-KO cells (Fig. 3j). Moreover, C351S mutation sensitized PFKM to the ATP and citrate inhibition in SKOV3-PFKM-KO cells (Fig. 3k). The potential of glycolysis contributes to drug resistance in cancer cells. DDP is one of the most commonly used drugs in ovarian cancer chemotherapy. PFKM-C351S mutation also sensitized the SKOV3 cells to DDP treatment (Fig. 3l). Protein structure analysis with PyMOL software showed that C351 of PFKM is located away from the binding sites of allosteric regulators such as ADP, AMP, citrate, ATP, and F-2,6-BP (Ser 377, Lys 678, Asn 341 for AMP and ADP binding, 88/89,118–121 for ATP binding, Arg 429/433 and Lys557/617, Asp591 for ATP and citrate inhibition, Arg 566/655 and His 661 sites for F-2,6-BP activation) (Fig. S3b). These results indicated that the S-nitrosylation of C351 promotes the tetramer formation of PFKM, contributing to NOS1-mediated enhancement of PFKM activity and tolerance of metabolites inhibition.

### S-nitrosylation of PFKM at Cys351 promoted glucose metabolism in ovarian cancer cells

**Fig. 4:**
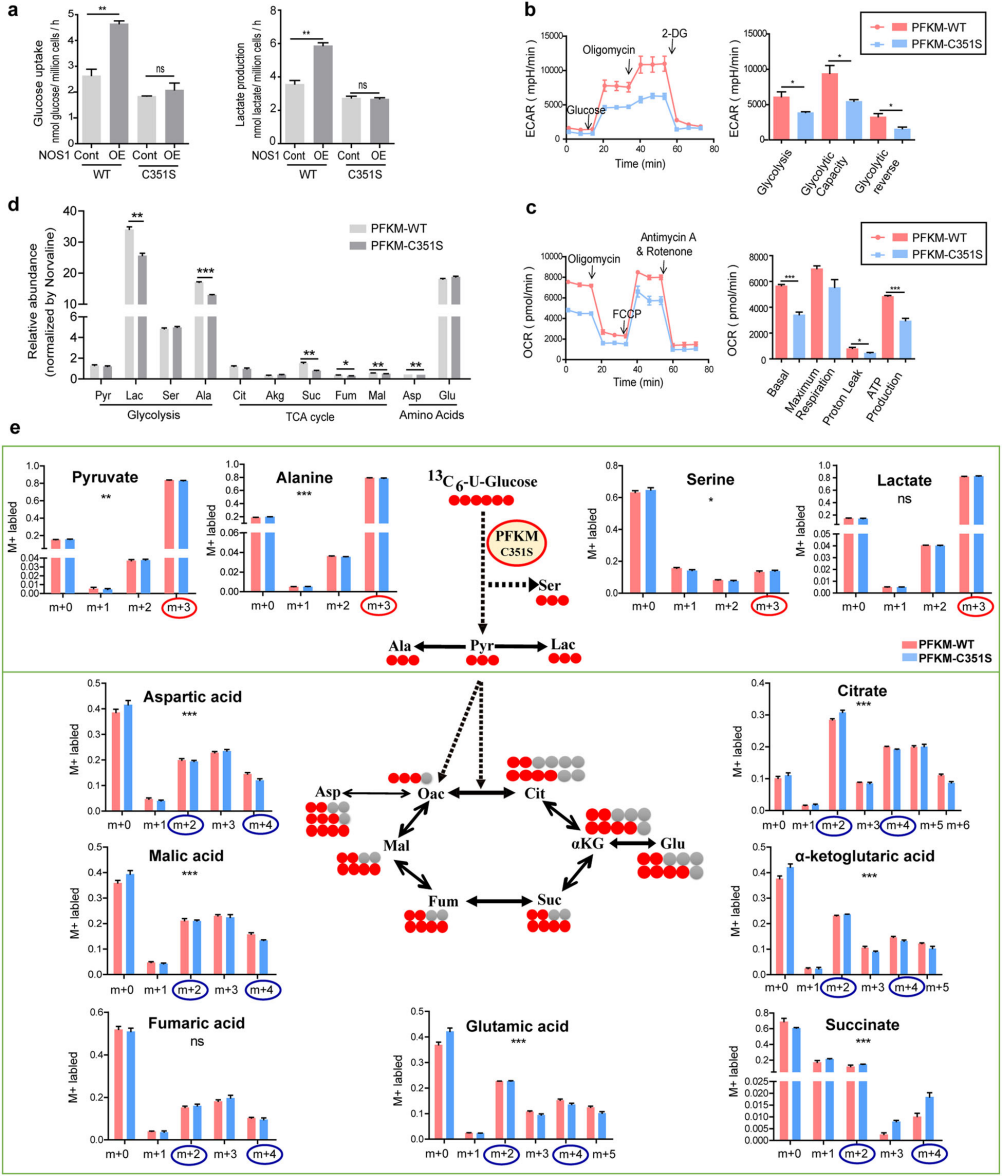
S-nitrosylation of PFKM at Cys351 promoted glucose metabolism in ovarian cancer cells. **a** Glucose uptake and lactate production of the SKOV3-PFKM-KO cells and SKOV3-PFKM-KO+NOS1 cells which were respectively reconstituted with Flag-PFKM-WT or Flag-PFKM-C351S. **b** Detection of cellular glycolytic capacity (ECAR) using Seahorse XF technology in PFKM-WT and PFKM-C351S SKOV3 cells. **c** Detection of cellular mitochondrial oxygen consumption (OCR) using Seahorse XF technology in PFKM-WT and PFKM-C351S SKOV3 cells. **d** Detect the total amount of glycolytic products, TCA cycle intermediate synthesis and amino acids in PFKM-WT or PFKM-C351S SKOV3 cells using GC-MS analysis technique. **e** The U-^13^C_6_-glucose label and the carbon atom orientation. Relative level of glycolysis (pyruvate, alanine, serine, and lactate) and TCA cycle (citrate, α-ketoglutaric acid, succinate, glutamic acid, fumaric acid, malic acid, and aspartic acid), determined by m + 2 or m + 4 labeling of metabolites from 13C-U-glucose in PFKM-WT (pink) and PFKM-C351S (blue) SKOV3 cells (*n* = 3) (*P < 0.05, **P < 0.01, ***P < 0.001 by two-way ANOVA).

To determine whether the S-nitrosylation of PFKM affects glucose metabolism and energy management, we evaluated the glycolytic rate, oxygen consumption, and glucose metabolites of the SKOV3 cells transfected with the C351S mutant. The effect of NOS1 on glucose uptake and lactic acid production was attenuated in SKOV3-PFKM-C351S cells (Fig. 4a). The cell energy metabolism tested by seahorse XF96 cell energy technology indicated the ECAR, a readout of lactate production, was reduced in PFKM-C351S cells (Fig. 4b). Moreover, the basal OCR, ATP production, and proton leak were all reduced in PFKM-C351S cells (Fig. 4c). We evaluated the relative abundance of glycolytic metabolites and TCA cycle metabolites in SKOV3-PFKM-C351S cells and SKOV3-PFKM-WT cells using the GC-MS technique. As expected, the relative abundance of glycolytic metabolites (lactic acid and alanine) and TCA cycle metabolites (succinic acid, fumaric acid, malic acid, and aspartic acid) reduced in SKOV3-PFKM-C351S cells (Fig. 4d).

The stable isotope-resolved metabolomics technique was used to determine the isotopomer distributions of metabolites and identify the metabolic pathways in which the S-nitrosylation of PFKM was involved. After ^13^C-U-glucose-labeling for 24 h, the metabolites were measured using the GC-MS analysis technique. Mass isotopomer distributions represent the relative abundance of ion fragments with a different number of ^13^C. The steady levels of metabolite and the metabolic pathways by testing the ^13^C-labeled metabolites enable us to analyze metabolic flux distribution. We observed that the total ^13^C-labeled pyruvate of glycolytic metabolites, citrate, α-ketoglutaric acid, malic acid, and glutamate in the TCA cycle were reduced in SKOV3-PFKM-C351S cells (Fig. S4a). Mass isotopomers m + 0, m + 1, m + 2, etc., refer to the ion fragments with zero, one, or two ^13^C, respectively. The proportions of the m + 3 isotopologue-labeled pyruvate, and alanine except for lactate (triply ^13^C-labeled or ^13^C_3_-), the metabolites directly derived from glucose, reduced in SKOV3-PFKM-C351S cells (Fig. 4e). The intermediates of the TCA cycle labeled with m + 2 and m + 4 isotopologues were derived from ^13^C-labeled glucose directly. Among them, the m + 2 and m + 4 represented the metabolites were in the first and second TCA cycle, respectively. The m + 2 isotopologue levels of citrate, α-ketoglutaric acid, glutamic acid, and fumaric acid increased, but their m + 4 isotopologue levels reduced in SKOV3-PFKM-C351S cells compared with SKOV3-PFKM-WT cells, indicating that PFKM-C351S promoted the TCA cycle to aggregate in the first cycle. Apparently, the mutation of PFKM Cys351 slowed the TCA cycle flux from glucose metabolites. These data suggested that PFKM-C351S mutation attenuated glycolytic activity and the mitochondrial oxidative phosphorylation (OXPHOS) capacity.

To further explore the effect of SNO-PFKM and NOS1 on cell proliferation, we generated two pairs of cell sub-strains, which stably expressed PFKM-WT or PFKM-C351S in SKOV3-PFKM-KO or SKOV3-PFKM-KO plus OE-NOS1 cells (PFKM-WT vs. OE-NOS1+PFKM-WT, PFKM-C351S vs. OE-NOS1+PFKM-C351S). PFKM-C351S mutation reduced cellular proliferation of OE-NOS1- and OE-NOS1 cells (Fig. S4b). Therefore, the enhancement of intracellular glucose metabolism by S-nitrosation of PFKM contributes to cellular proliferation.

### PFKM C351S mutation abolished the NOS1 promotion of tumor growth in mouse xenograft models

**Fig. 5:**
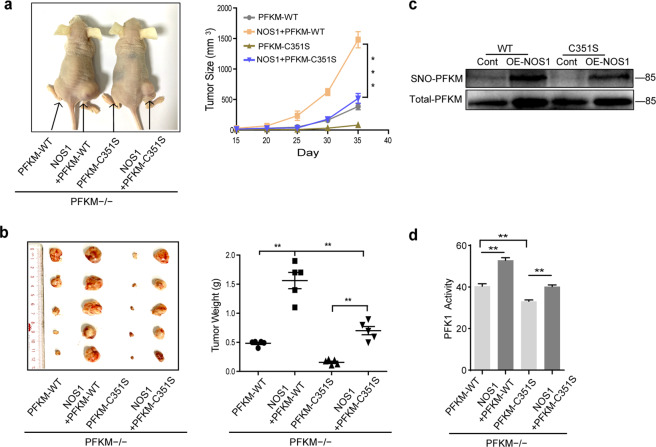
PFKM C351S mutation abolished the NOS1 promotion of tumor growth in mouse xenograft models. **a** Representative images showing the effect of SKOV3-PFKM-C351S on tumor size (left); statistical analyses showing the effect of SKOV3-PFKM-C351S on tumor size (right). **b** Tumor weight in nude mice which injected the SKOV3-PFKM-WT, SKOV3-NOS1+PFKM-WT, SKOV3-PFKM-C351S, and SKOV3-NOS1+PFKM-C351S cells, respectively (*n* = 5). **c** Western blot detection of the content of SNO-PFKM protein in the SKOV3-PFKM-WT, SKOV3-NOS1+PFKM-WT, SKOV3-PFKM-C351S, and SKOV3-NOS1+PFKM-C351S tumor tissues. **d** The activities of PFK1 were measured in the SKOV3-PFKM-WT, SKOV3-NOS1+PFKM-WT, SKOV3-PFKM-C351S, and SKOV3-NOS1+PFKM-C351S tumor tissues. **P < 0.01, Student’s *t-*test.

The xenograft model of the subcutaneous tumor was generated with SKOV3-PFKM-WT vs. SKOV3-NOS1+PFKM-WT and SKOV3-PFKM-C351S vs. SKOV3-NOS1+PFKM-C351S cells. PFKM-C351S mutation reduced the size and weight of tumors compared to those of PFKM-WT tumor, and NOS1 promotion of tumor reduced by PFKM-C351S mutation (Fig. 5a, b). Moreover, consistent with the in vitro experiments, PFKM-C351S reduced the content of S-nitrosated PFKM and the activity of PFK1 in SKOV3 tumor extracts (Fig. 5c, d).

Our previous studies showed that NOS1 can promote lung metastasis of melanoma^32^. To explored the effect of SNO-PFKM on the metastasis potential of cancer cells and the tumor infiltration of immune cells in C57BL/6 mice, the B16-F10 melanoma cells transfected with PFKM-WT or C351S after PFKM knocked out were injected into mice through the tail vein. As shown in Fig. S5a and S5b the number of lung nodules in the B16-PFKM-C351S group was significantly less than that in the B16-PFKM-WT group. The lung weight with B16-PFKM-C351S tumors was substantially less than that in B16-PFKM-WT. Moreover, the mean survival time of B16-PFKM-C351S mice was significantly longer than the B16-PFKM-WT mice (27 days for PFKM-C351S and 22.5 days for PFKM-WT) (Fig. S5c). The infiltration of immune cells in tumor tissues was detected by the flow cytometry technique. As shown in Supplementary Fig. S5e, f the subsets of CD11b+F4/80+ macrophages increased in tumor tissues of B16-PFKM-C351S compared to that of B16-PFKM-WT. The number of activated T cells (CD25+CD3+ T cells) elevated slightly in B16-PFKM-C351S tumors. Therefore, the enhancement of intracellular glucose metabolism by S-nitrosation of PFKM contributes to cellular proliferation and tumor metastasis.

## Discussion

Posttranslational modification affects the spatial structure and oligomers formation of enzymes^36^. It is a pivotal mechanism in regulating glycolysis flux to support the rapid proliferation of cancer cells. Phosphorylation of PFKP-Y64 via AKT activation enhances PFK1 activity and the Warburg effect^8^. The glycosylation of PFKL at S529 results in the downregulation of its catalytic activity and redirects glucose flux through the pentose phosphate pathway for the anti-apoptosis of tumors^37^. Here we demonstrated that NOS1 induces PFKM S-nitrosation at Cys351 to stabilize tetramer of PFK1 and increase its activity, leading to the increase of the glycolysis in ovarian cancer cells. This modification interferes with the feedback regulation of glycolysis and imposes tumor cells with a high glycolysis flux. Whether other enzymes in glycolysis were known to be S-nitrosylated by NOS1 or other NOSs needs confirmation by further experiments. Nitric oxide has been well established as a modulator of cellular bioenergetics in metabolism shifting to glycolysis by inhibiting cellular respiratory function^38^. Thus, we provided a distinct mechanism of NO in regulation on metabolism conversion in tumors. S-nitrosation of PFKM and cellular respiration inhibition may work together for the endogenous NO regulation on the metabolism switch of cancer.

The activity of PFK1 responds to extracellular nutrients and stimulation of growth signals, thus regulating glycolysis rate for cellular biological functions. Tumor cells have a high glycolytic flow, generating amounts of metabolism intermediates for anabolism and cellular proliferation. PFKM expression in tumors is associated with poor prognosis. It was reported that a shorter isoform of PFKM (47 kDa) was detected in some tumorigenic cell lines and resistant to citric acid and ATP inhibition^39^. PFKM bound with Calmodulin (CaM) forms active dimers less susceptible to allosteric inhibition^40^. Our findings indicated that S-nitrosylated PFKM enhanced the stability of PFKM active tetramer for the tolerance of a high level of ATP and citrate in cancer cells. PFKM expression is positively correlated with the progressive stages of ovarian cancer. It has documented that PFK1 clusters with other glycolytic enzymes form “glycosome” for high efficiency of glucose metabolism. In our S-nitrosoproteomic profile, the primary molecular function of S-nitrosation proteins is enriched in protein binding. S-nitrosylation of PFKM may participate in the formation of glycosome in tumor metabolism alteration.

NOS1 is highly expressed in cancer tissues and usually promotes tumor progression by synthesizing a low level of NO and enhancing cell proliferation, anti-apoptosis, and migration. Three isozymes of NOSs: neuronal NOS (NOS1), inducible NOS (NOS2), and endothelial NOS (NOS3), all contribute to tumor progression through distinct effects of biological regulation on tumorigenesis. Unlike the other two isoforms, inducible NOS (NOS2) expression is induced by inflammatory cytokines, hypoxia, and oxidative stress^41^. NOS3 expression is low in ovarian cancer. PFK activity is exclusively affected by NOS1 but not by NOS3 or NOS2 (ref. ^42^). NOS1 has a PDZ functional domain and specifically binds to target proteins, thereby selectively modifying characteristic sites and regulating special signal pathways or biological processes. Thus, S-nitrosylation of PFKM may be dominated by NOS1 and be critical for NOS1 induced tumor promotion in various types of cancer.

The accumulation of lactic acid from glycolysis makes an immunosuppressive effect on immune cells like dendritic cells and cytotoxic T cells in tumor microenvironment^43,44^. Here, S-nitrosylated PFKM by NOS1 promoted lactic acid production extracellularly and intracellularly, and reduced macrophages’ infiltration in tumor tissues. Moreover, the S-nitrosoproteomic profile of ovarian cancer cells indicated multiple proteins of metabolism processes, and immune responses were targeted by S-nitrosylation modification. The comprehension of the molecular mechanisms underlying the regulation of metabolism and immune by NOS1 induced S-nitrosylation may lead to new therapeutic strategies to control tumor progresses.

## Supplementary information

Supplemental information

Figure S1

Figure S2

Figure S3

Figure S4

Figure S5

SNO-peptide

## Data Availability

All participants agree to publish all data.
