# Supplementary material for: The role of S-nitrosylation of PFKM in regulation of glycolysis in ovarian cancer cells
Source: Cell Death Dis. 2021 Apr 15;12(4):408. doi: 10.1038/s41419-021-03681-0 (PMC8050300; doi:10.1038/s41419-021-03681-0)
Supplement: Supplementary file 1 — Supplemental information [file 41419_2021_3681_MOESM1_ESM.docx]

**Supplemental information**

**
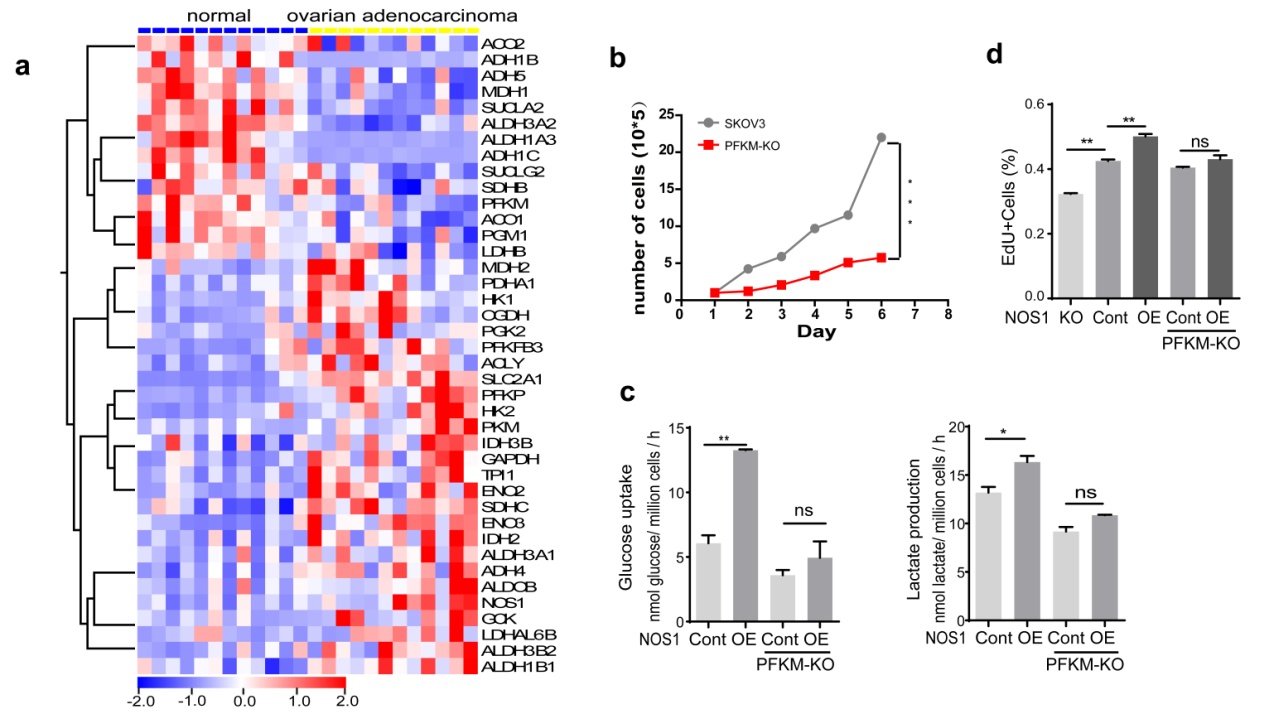
**

**Figure S1 related to Figure 1.** **The key components in glycolysis pathway are highly S-nitrosylated in ovarian cancer cells. a** Heat map displayed the genes (rows) differentially expressed in 12 ovarian normal epithelia and 12 malignant epithelial samples (columns). **b** Cell counting test to detected the growth rate of the SKOV3 cells and SKOV3-PFKM-KO cells (n=3) (****P*<0.001 by one-way ANOVA). **c** Glucose uptake and lactate production of OE-NOS1 vs. control SKOV3 cells and OE-NOS1 vs. control SKOV3 cells which were knocked out PFKM (PFKM-KO) cells (n=3). **d** The cell proliferation rates were detected in NOS1-KO cells, control SKOV3 cells, OE-NOS1 cells, and OE-NOS1 vs. control SKOV3 cells which were knocked out PFKM (PFKM-KO) cells by EdU cell proliferation assay. Each value represents the mean ± SD in three independent experiments. * *P* <0.05, ***p* < 0.01, ns, not significant, Student’s t test.


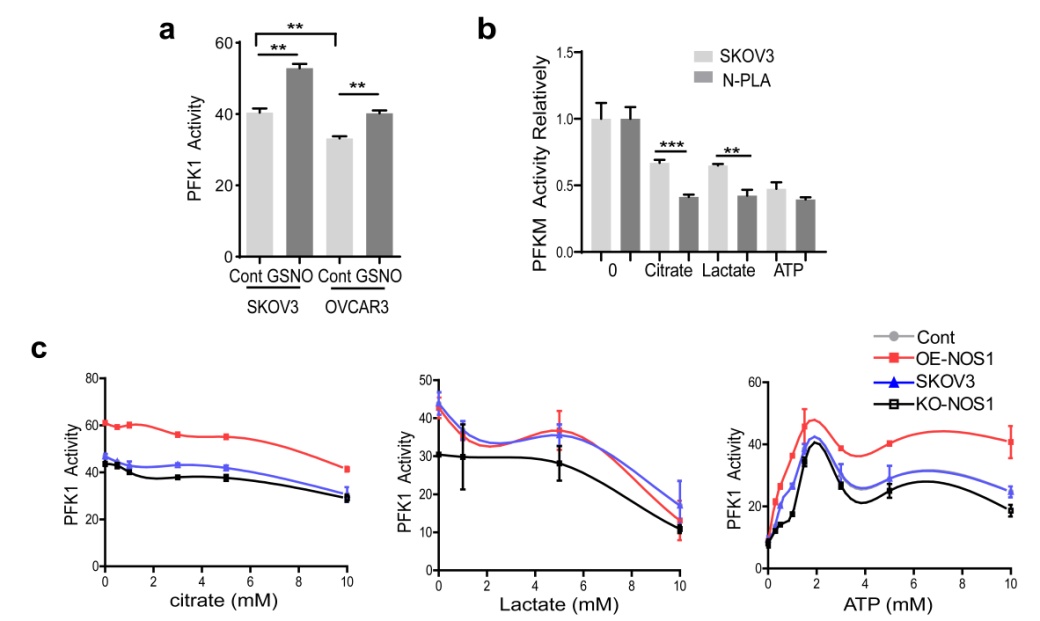


**Figure S2 related to Figure 2.** **NOS1 enhances its activity through S-nitrosation of PFKM. a** The enzymatic activities of PFK1 were detected in the SKOV3 and OVCAR3 cells which were treated with PBS (Cont) or GSNO（1mM）for 30min (n=3). **b** The relative activity of purified PFKM under different concentrations of Citrate (5mM), Lactate (5mM) and ATP (5mM) in SKOV3 cells and SKOV3 cells treated with N-PLA (100uM) for 48h (n=3). **c** The activity of total PFK1 under different concentrations of Citrate (left), Lactate (middle) and ATP (right) in SKOV3 vs. NOS1-KO cells and SKOV3-OE-NOS1 vs. control cells. Data represent the means ± SD. of three independent experiments. ** *P* <0.01, ****P* <0.001, Student’s t test.


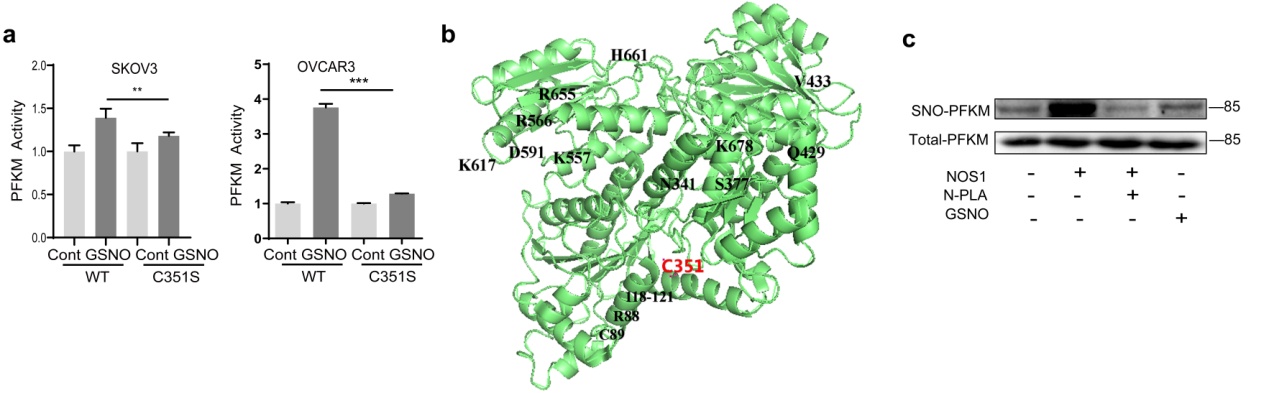


**Figure S3 related to Figure 3. NOS1 induced S-nitrosylation of PFKM at Cys351.** **a** The activities of PFKM were measured in SKOV3 and OVCAR3 cells, which were respectively reconstituted with PFKM-WT or PFKM-C351S, with PBS (Cont) or GSNO（1mM） for 30min. All results were normalized with their own control group. Each value represents the mean ± SD in five independent experiments. ** *P* <0.01, ****P* <0.001, Student’s t test. **b** PyMOL software analyzed the relationship between the S-nitrosation site and other metabolite binding sites. **c** Biotin switch then purified all S-nitrosylated proteins in OVCAR3 cells using streptavidin agarose resins. Western blot detected the content of S-nitrosation-modified PFKM (SNO-PFKM) protein in the OVCAR3 cells, OE-NOS1 cells, the cells which were treated with GSNO（1mM）for 30min and the OE-NOS1 cells treated with NOS1 specific inhibitor N-PLA (100 μM) for 48 h.


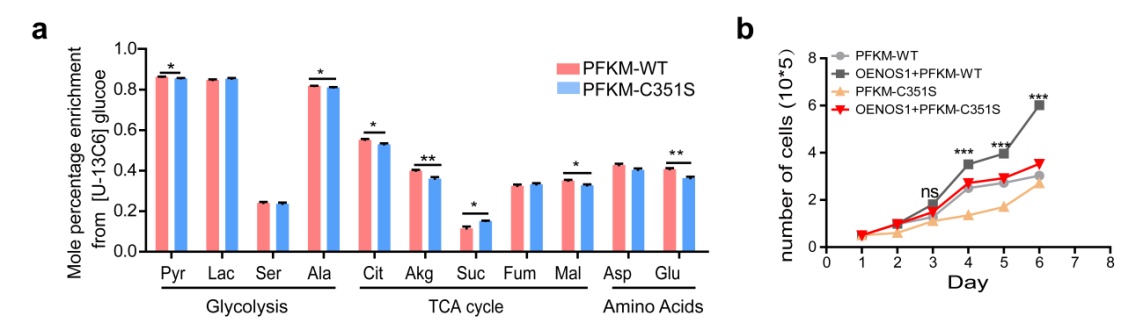


**Figure S4 related to Figure 4.** **S-nitrosylation of PFKM at Cys351 promoted glucose metabolism in cancer cells. a** The mole percentage enrichment from [U-^13^C_6_] glucose in PFKM-WT (pink) and PFKM-C351S (blue) SKOV3 cells (n=3). **b** Cell counting test to detect the growth rate of the above four cells (SKOV3-PFKM-WT, SKOV3-OENOS1+PFKM-WT, SKOV3-PFKM-C351S, and SKOV3-OENOS1+PFKM-C351S) ns, not significant; * *P* <0.05, ** *P* <0.01,****P* <0.001, Student’s t test.

**
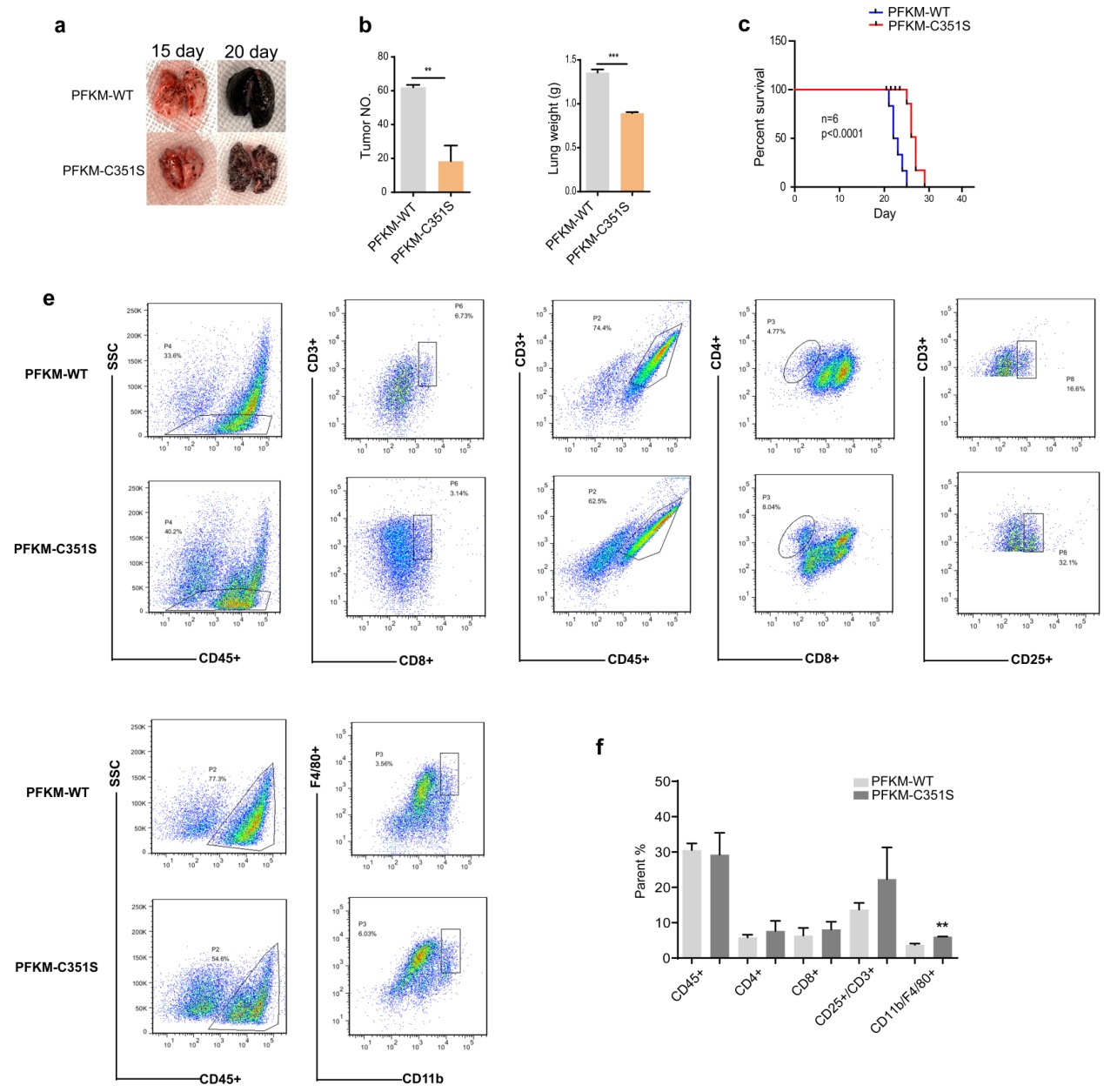
**

**Figure S5 related to Figure 5. PFKM C351S mutation abolished the NOS1 promotion of tumor growth in mouse xenograft models. a**. **b** When signs of metastatic distress and pulmonary metastasis were confirmed by histology, the mice were sacrificed on day 15 and the number of lung nodules was counted. On day 20, the mice were sacrificed and the lungs were weighed. **c** Long rank analysis of mouse survival rates (n=6). ****P*<0.0001 by Log-rank (Mantel-Cox) Test. **e** Representative flow cytometry plots were shown. **f** CD45, CD4, CD25, CD8 and CD11b-F4/80 followed by flow cytometry analysis. Quantification is of data from 3 animals in each group.

**Table S1: Endogenously S-nitrosylated proteins in glycolysis pathway.**

| **Master Protein Accessions** | **Gene Symbol** | **Description** | **Positions in Master Proteins** | **Annotated Sequence** |
| --- | --- | --- | --- | --- |
| **P19367** | **HK1** | **Hexokinase-1** | **Cys632** | **ATD CVGHDVVTLLR** |
| **P08237** | **PFKM** | **ATP-dependent 6-phosphofructokinase, muscle type** | **Cys351** | **LPLMECVQVTK** |
| **Q01813** | **PFKP** | **ATP-dependent 6-phosphofructokinase, platelet type** | **Cys112**  **Cys656**  **Cys718** | **AACNLLQR**  **GVFDCR**  **FTTDDSICVLGISK** |
| **P04075** | **ALDOA** | **Fructose-bisphosphate aldolase A** | **Cys127**  **Cys232**  **Cys339**  **Cys344** | **VNPCIGGVILFHETLYQK YASICQQNGIVPIVEPEILPDGDHDLK RALANSLACQGK**  **CPLLKPWALTFSYGR** |
| **P60174** | **TPI1** | **Triosephosphate isomerase** | **Cys79**  **Cys87**  **Cys104**  **Cys255** | **VPADTEVVCAPPTAYIDFAR**  **DCGATWVVLGHSER**  **IAVAAQNCYK**  **IIYGGSVTGATCK** |
| **P04406** | **GAPDH** | **glyceraldehyde-3-phosphate dehydrogenase** | **Cys156**  **Cys247** | **IISNASCTTNCLAPLAK**  **VPTANVSVVDLTCR** |
| **P00558** | **PGK** | **phosphoglycerate kinase 1** | **Cys99** | **DCVGPEVEK** |
| **P18669** | **PGAM** | **Phosphoglycerate mutase 1** | **Cys153** | **YADLTEDQLPSCESLK** |
| **P06733** | **ENO** | **alpha-enolase** | **Cys119**  **Cys357** | **FGANAILGVSLAVCK**  **VNQIGSVTESLQACK** |
| **P14618** | **PKM** | **Pyruvate kinase** | **Cys49**  **Cys226**  **Cys326**  **Cys358**  **Cys423/424**  **Cys548** | **NTGIICTIGPASR**  **CDENILWLDYK**  **AGKPVICATQMLESMIK**  **AEGSDVANAVLDGADCIMLSGETAK**  **CCSGAIIVLTK**  **GIFPVLCK** |
| **P00338** | **LDHA** | **L-lactate dehydrogenase A** | **Cys214**  **Cys322** | **LGVHPLSCHGWVLGEHGDSSVPVWSGMNVAGVSLK**  **DDVFLSVPCILGQNGISDLVK** |
| **P07195** | **LDHB** | **L-lactate dehydrogenase B** | **Cys164** | **VIGSGCNLDSAR** |
