# Supplementary figures and images for: The role of S-nitrosylation of PFKM in regulation of glycolysis in ovarian cancer cells
Source: Cell Death Dis. 2021 Apr 15;12(4):408. doi: 10.1038/s41419-021-03681-0 (PMC8050300; doi:10.1038/s41419-021-03681-0)

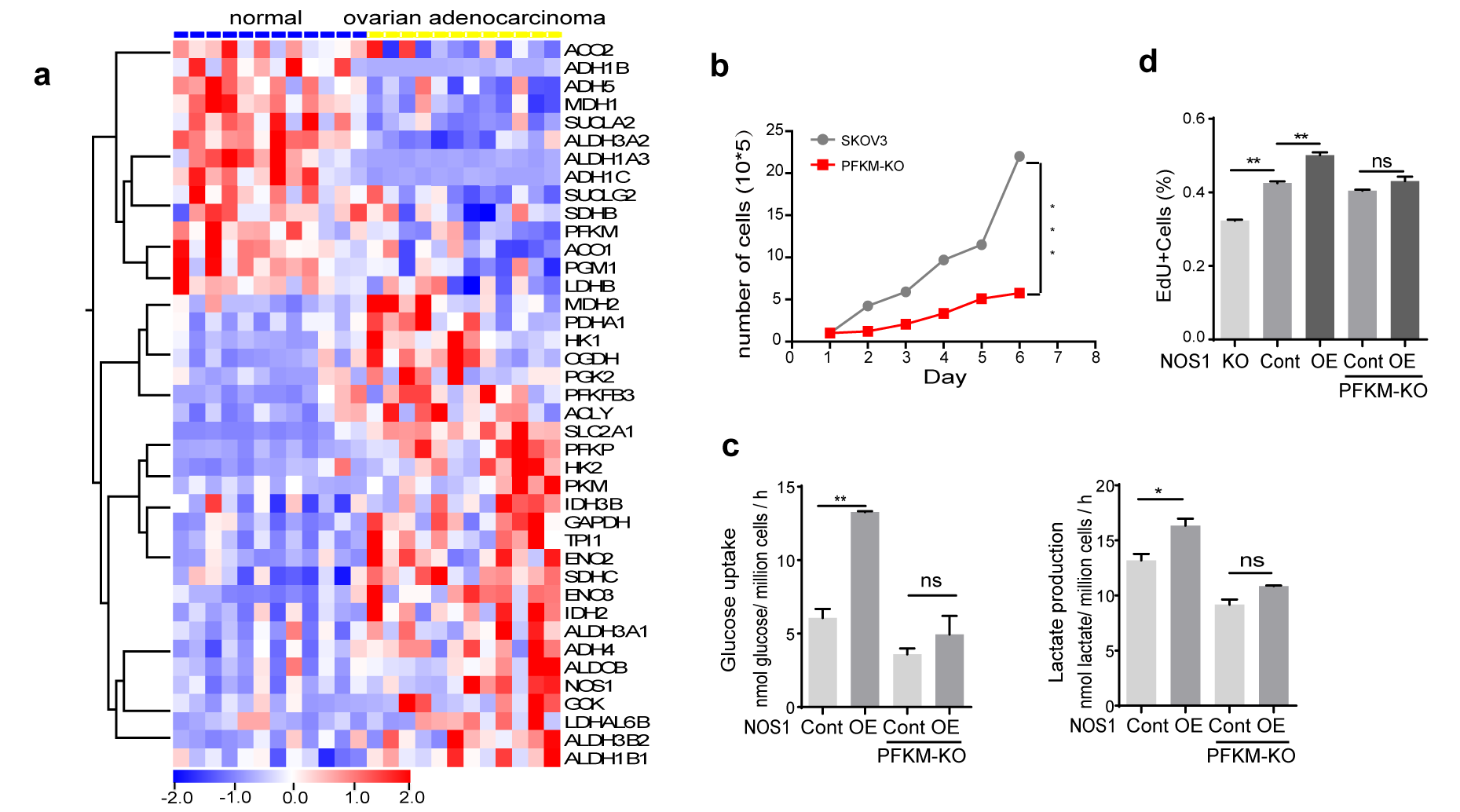

Supplement: Supplementary file 2 — Figure S1 [file 41419_2021_3681_MOESM2_ESM.tif]

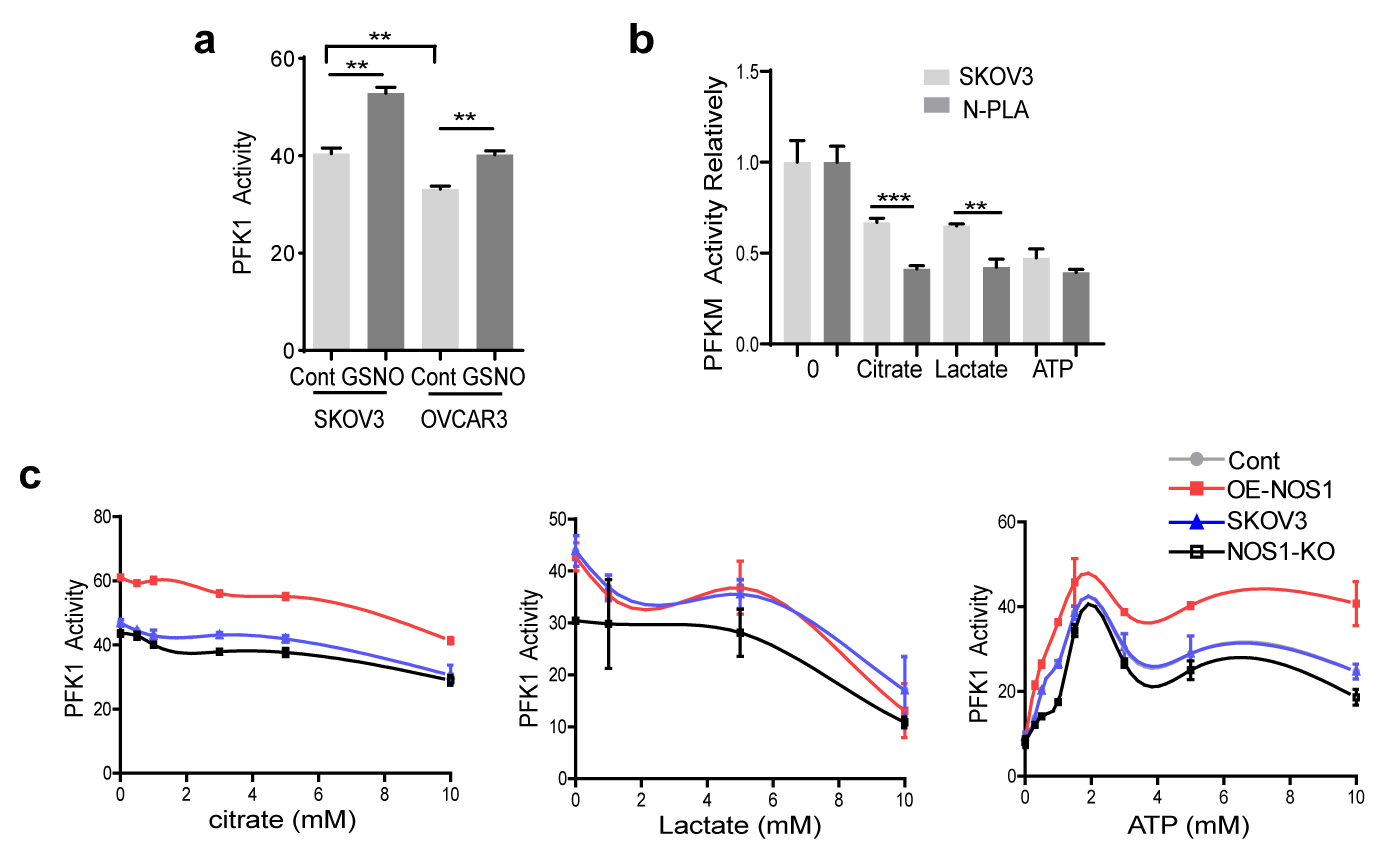

Supplement: Supplementary file 3 — Figure S2 [file 41419_2021_3681_MOESM3_ESM.tif]

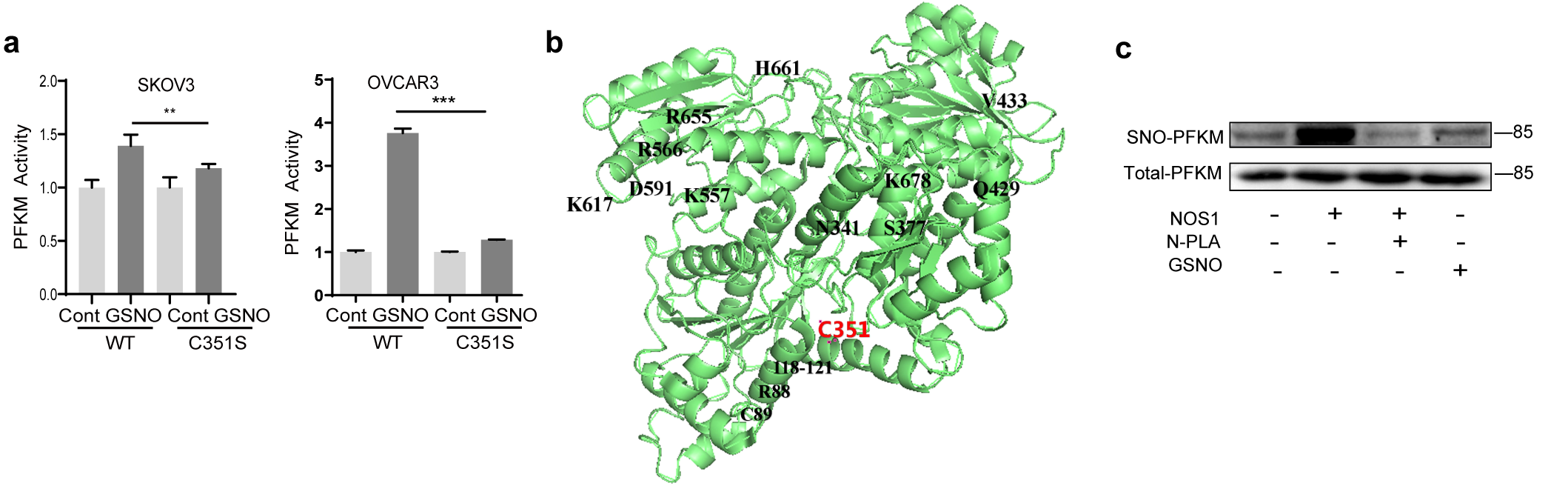

Supplement: Supplementary file 4 — Figure S3 [file 41419_2021_3681_MOESM4_ESM.tif]

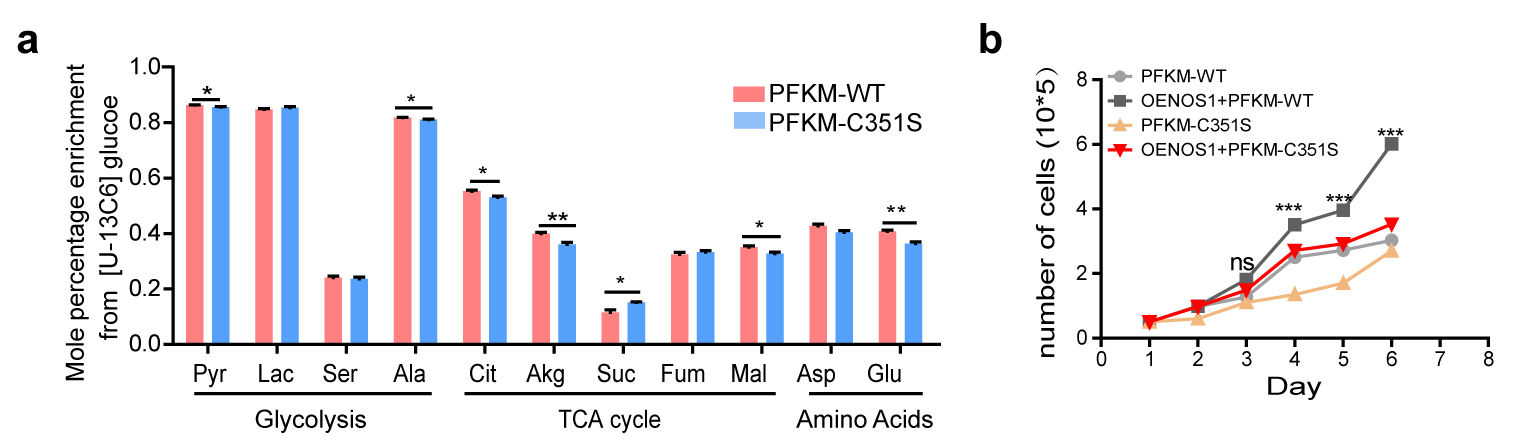

Supplement: Supplementary file 5 — Figure S4 [file 41419_2021_3681_MOESM5_ESM.tif]

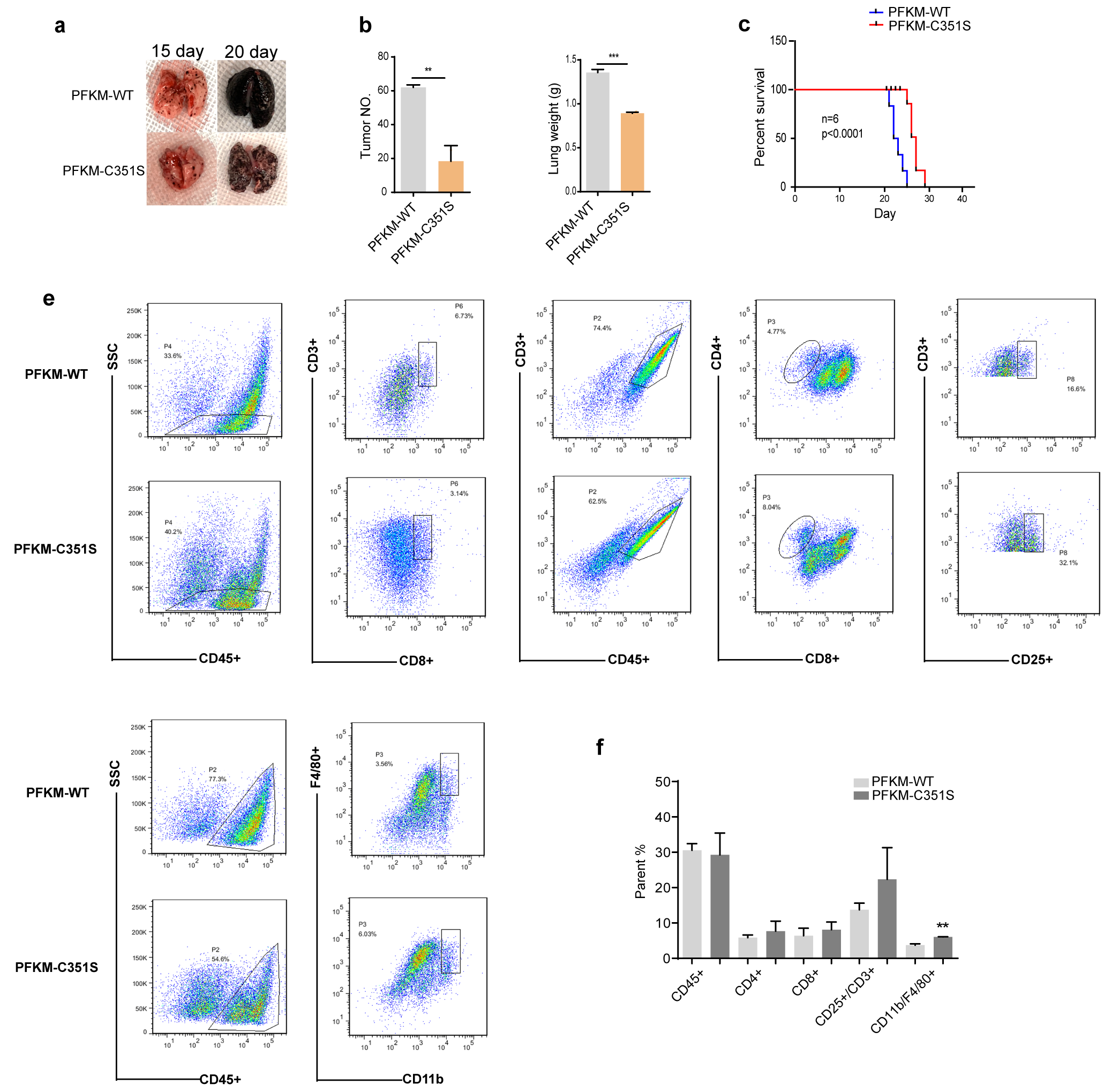

Supplement: Supplementary file 6 — Figure S5 [file 41419_2021_3681_MOESM6_ESM.tif]
